# Diabetes care and service access among elderly Vietnamese with type 2 diabetes

**DOI:** 10.1186/1472-6963-13-447

**Published:** 2013-10-29

**Authors:** Mary C Carolan-Olah, Angie Cassar, Regina Quiazon, Sean Lynch

**Affiliations:** 1 Inner North West Melbourne Medicare Local, Level 1, 369 Royal Parade, Parkville, Victoria, Australia; 2 Inner North West Primary Care Partnership, 11 Glenlyon Road, Brunswick, Victoria, Australia; 3 College of Health and Biomedicine, Victoria University, McKechnie Street, St Albans, Victoria, Australia; 4 Multicultural Centre for Women’s Health, Suite 207, Level 2, Carringbush Building, 134 Cambridge Street, Collingwood, Victoria, Australia

## Abstract

**Background:**

Vietnamese patients are disproportionately represented in type 2 diabetes mellitus statistics and also incur high rates of diabetes complications. This situation is compounded by limited access to health care. The aim of this project was to gain a deeper understanding of the difficulties Vietnamese patients experience when accessing services and managing their type 2 diabetes mellitus, and to identify factors that are important in promoting health service use.

**Methods:**

Three focus groups with 15 Vietnamese participants with type 2 diabetes mellitus, 60 to >70 years of age, were conducted in Vietnamese. Open-ended questions were used and focussed on experiences of living with diabetes and access to healthcare services in the Inner Northwest Melbourne region. Audio recordings were transcribed and then translated into English. Data were analysed using a thematic analysis framework.

**Results:**

Findings indicate four main themes, which together provide some insight into the experiences of living with diabetes and accessing ongoing care and support, for elderly Vietnamese with type 2 diabetes. Themes included: (1) the value of being healthy; (2) controlling diabetes; (3) staying healthy; and (4) improving services and information access.

**Conclusions:**

Participants in this study were encouraged to adhere to diabetes self-management principles, based largely on a fear of medical complications. Important aspects of healthcare access were identified as; being treated with respect, having their questions answered and having access to interpreters and information in Vietnamese. Attention to these details is likely to lead to improved access to healthcare services and ultimately to improve glycemic control and overall health status for this community.

## Background

Diabetes is one of the leading chronic diseases in Australia, and this condition was responsible for approximately 6% of the total burden of disease in 2003 [1]. Type 2 diabetes mellitus (T2DM), which is the most common type of diabetes in Australia (87%), accounts for 92% of that burden [1]. As such, type 2 diabetes care constitutes a substantial cost for the healthcare system [1]. This form of diabetes, which mostly affects older adults is linked to obesity and sedentary lifestyle. It is also strongly associated with a number of serious health complications, which occur as a result of micro and macro vascular changes [1]. Moreover, the prevalence of T2DM has increased dramatically in the past two decades and this condition is now considered one of the greatest health challenges facing healthcare providers in the 21^st^ century [2,3]. Rates in Australia have more than doubled during this period [4] and evidence suggests that this prevalence is underestimated at the same time as rates of T2DM continue to rise [1,5].

Risk factors for T2DM include obesity, sedentary lifestyle and poor diet, and ethnicity from world areas of high diabetes prevalence, such as: South East Asia (principally Vietnam and Philippines); the Middle East [6]; South Europe [7] and South Asia (Indian subcontinent) [8]. In this study, we were particularly interested in the Vietnamese population, which constitutes one of the most populous groups of migrants in Australia [9] and in 2011, the number of Vietnamese born in Australia was 180,000 [10]. Vietnamese individuals also make up a sizeable percentage of the population in our local area [11]. There is good quality evidence to suggest that Vietnamese populations are more likely to develop T2DM, compared to Caucasian populations [12-14], and to do so at lower BMI levels [15]. The extent of this increased prevalence is evident in a recent study conducted by Ta et al., (2010), in Ho Chi Minh city, Vietnam, where rates of T2DM were found to be 10.8% of men and 11.7% of women, compared to approximately 7% in Australian populations [1]. Additionally, while the average BMI of Caucasians with T2DM is approximately 26–27.5/m^2^, [16], the average BMI of Vietnamese T2DM patients is around 23 kg/m^2^[17]. As well as occurring at lower BMI levels, rates of T2DM increase more rapidly in the Vietnamese, with even modest increases in BMI [12]. This overall increased prevalence of T2DM is thought to be linked to greater levels of abdominal and visceral adiposity in Vietnamese individuals [17,18] compared to other ethnicities.

Nonetheless, despite this high prevalence of T2DM and large numbers of Vietnamese migrants in Australia, research on diabetes in Vietnamese populations in Australia, remains limited. A small body of literature was located [14,19-21] and Tran et al., [14,21], for example, investigated both the prevalence of type 2 diabetes among the Vietnamese born population in New South Wales, and the impact of acculturation on type 2 diabetes rates. Similar to international studies, these authors found much higher rates of T2DM in the Vietnamese born population compared to the Australian born population (14.7% vs 7.4) [14]. Rose and Harris [19] who conducted qualitative interviews among Vietnamese speaking participants with T2DM found that this group experienced a number of challenges with their diabetes self-management, including poor social support and minimisation of diabetes by friends and family. On the other hand, Swerisson et al., [20] who investigated the efficacy of a chronic disease self-management program, among different ethnic groups with a range of chronic diseases, found that the Vietnamese speaking participants responded well to the intervention, reporting significantly higher levels of self-efficacy and self-rated health, compared to the control group. Although there is no clear consensus in the literature, the suggestion is that that the Vietnamese encounter a number of challenges in accessing services and managing their diabetes. This is commensurate with evidence that indicates that poorer access to health services is common among culturally and linguistically diverse (CALD) communities [22]. For the Vietnamese, this situation is compounded by factors such as: low socio economic status [23], poorer general education [24] and low English language skills [25]. Together these factors limit health care access and lead to poorer health literacy, or a limited ability to read and comprehend health information [26,27].

For all these reasons, this project aimed to explore the health consumer experience of diabetes care and self-management in the Inner North West Melbourne region. In this area, which is one of Melbourne’s most socially and economically disadvantaged suburbs [28], population demographics indicate a very diverse population with > 20% of inhabitants having been born overseas, many of whom have come from Vietnam. Consumers with T2DM were recruited from two of the most prevalent ethnic groups in the region–Italian and Vietnamese [11]. This paper reports on the Vietnamese focus groups, which were conducted in Vietnamese. Our project is different from earlier studies as it sought out and specifically recruited elderly migrant health consumers with limited English skills. These groups are underrepresented in research [29-32], and reasons for this underrepresentation includes: an unwillingness on the part of would be participants, due to language difficulties, a lack of knowledge about research studies/consent processes and a lack of understanding about health promotion [32]. Difficulties reported by researchers include difficulties of access to minority communities and high attrition rates from research projects [33]. At the same time as being underrepresented in research, these groups suffer disproportionate rates of diabetes complications [34] and service access difficulties [22]. As such, the contribution of elderly migrant health consumers with limited English skills, in this project, was seen as an important aspect of health service planning and design in the region.

## Methods

A qualitative approach was used and three focus groups, with 15 Vietnamese participants, were conducted in Vietnamese. Pre-determined open-ended questions were used and focussed on participants’ experiences of living with T2DM, and the challenges they faced when accessing care for their type 2 diabetes. Prior to commencement, consultation with an expert panel occurred. This group included membership from acute and community health services and consumer engagement personnel, not for profit health agencies, and consumer representatives. The panel guided question formulation for the focus group discussions (see Table 1).

**Table 1:** Questions for focus group

**Category**	**Questions for focus groups**
**Meanings, beliefs, personal values**	**1. What does feeling healthy mean? Why?**
	**2. How has diabetes affected your health?**
	**3. How has it impacted on your day-to-day life?**
	**4. What made you take action in managing your diabetes?**
	**5. What is most difficult about managing your diabetes?**
**Access to information and services**	**1. Who do you currently see/go to for your diabetes care?**
	**2. What are your expectations of health service providers/GP/person providing you with diabetes care? Are you happy with the service/care you receive? Why? Why not?**
	**3. How did you or do you find out about services and information about diabetes?**
	**4. Do you need more information? On what topics?**
	**5. Whose job is it to inform the patient about all the service options (for multidisciplinary holistic care) which may be available to them? GP? Practice Nurse? Diabetes Educator? Specialist?**
	**6. Do you have difficulties accessing your local diabetes services? Why? What would help you access those services?**
	**7. Would you like to receive information in your own language?**
	**8. Have you ever been offered information in your own language? If so, types of information offered and from where?**
**Feedback and suggestions**	**1. What advice would you give someone who has type 2 diabetes?**
	**2. Some people are reluctant to access services. What do you think would assist these people to access diabetes care?**

This method of data collection was chosen as a useful means of collecting a relatively large amount of information, from a number of individuals, in a relatively short period of time [35]. The focus group method is also considered particularly useful when participants share certain characteristics relevant to a particular study, as in this case, when all participants are Vietnamese, have developed T2DM and come from the Inner North West Region of Melbourne. A particular strength of this approach is its social orientation, which tends to promote group interaction and to allow the interviewer to probe and elicit more in-depth information [35]. Focus group data was collected by bilingual focus group facilitators and scribes employed by the Multicultural Centre for Women’s Health (MCWH). Staff at this centre, are specifically trained in cultural sensitivity. Focus group facilitator training was also included, as follows: focus group delivery training (half a day), which included up-skilling from health education delivery to focus group delivery. Focus groups were recorded in Vietnamese and translated into English. Ethics approval for the study was granted by the Victoria University Human Research Ethics committee (HRE13-003).

### Recruitment and consent procedures

Bilingual recruitment flyers were posted at culturally specific community based organisations, in community media (newspapers and radio announcements), community health centres and general practitioner waiting rooms in the area. All recruitment, consent and evaluation materials were provided in Vietnamese and documents were professionally translated by NAATI (National Accreditation Authority for Translators and Interpreters) accredited interpreters, who are trained in cultural sensitivity. Bilingual administrative staff explained the project and provided researcher details. Individuals who were interested in participating were provided with details about the purpose of the study along with likely time requirements and the voluntary nature of participation. They were also given an information sheet to take home and discuss with family members. Bilingual opportunity was provided for interested individuals to ask questions and voice concerns. Participants who indicated their willingness to participate in the focus group sessions, had their contact details forwarded to the Multicultural Centre for Women’s Health staff, who were engaged to undertake the focus group sessions. Individuals who agreed to participate, had their written consent recorded prior to participation. A total of 30 participants were approached and 15 agreed to participate.

### Data analysis

De-identified focus group data was provided to research team members and data were analysed using a thematic analysis framework, informed by Burnard’s [25] approach. The following steps were employed:

• Familiarisation with the transcripts. This was achieved through reading and re-reading the transcripts until researchers were confident that they were familiar with the content

• Seeking out common concepts, ideas and perceptions

• Classification of concepts into units of meanings

• Ensuring reliability of analysis. Co-researchers independently reviewed the data and drew up lists of concepts and ideas

• Meeting of co-researchers and discussion on themes until agreement was met

• Merging of categories and headings as analysis proceeded

• Returning to the data to seek alternate meanings for the emerging themes [36], [p.462-463]

## Results

Demographics revealed that participants were predominantly female (11/15) and were aged in excess of 60 years, with the bulk of participants aged more than 70 years (n = 11). Duration of T2DM varied from more than one year (n = 6) to more than 5 years (n = 9). Participants had been resident in Australia for a minimum of 10 years. English language proficiency was low and all required an interpreter. Highest educational level was high school certificate (n =2) although the majority (n = 13) had not completed high school (see Table 2).

**Table 2:** Demographics of the focus group participants

**Cultural background**	**Number of participants**	**Gender**		**Length of time in Australia**		**Age**		**Time since diagnosis of diabetes**	
	**Total over 3 groups**	**Male**	**Female**	**<10 years**	**>10 years**	**Range**	**n**	**Time**	**n**
**Vietnamese**	**15**	**4**	**11**	**0**	**15**	40-49 years	0	<6 months	**0**
						50-59 years	0	< 1 year	**0**
						60-69 years	4	1-2 years	**2**
						≥70 years	11	2-5 years	**4**
						>5 years	9		

### Themes

Overall, four main themes emerged from the data analysis, and together they shed some light on participants’ experiences of living with diabetes and accessing ongoing care and support. Themes include: (1) the value of being healthy; (2) controlling diabetes; (3) staying healthy; and (4) improving services and information access. Each of these themes is described below.

### The value of being healthy

Participants in this study rated their health as very important, and more essential to life than financial wealth. Most equated health with having the physical capacity to do whatever they wished to do, which included exercising and undertaking simple chores, such as mowing the lawn. This broad definition of health included both physical and psychological elements, such as having energy, sleeping and eating well, and feeling happy and cheerful. Some illustrative quotes follow:

Health is golden, so good health is a priority not only for the elderly but also for everyone. For me, being healthy is more important than money.

Being healthy is when one is able to eat well, sleep well, and be happy and comfortable.

Being healthy means not feeling tired when performing daily tasks.

To have good health we must, have a diet, chat funny stories and don’t think so much.

### Controlling diabetes

In the second theme, the effect of diabetes on the individual’s life was described in stark detail and participants referred to their diagnosis using a range of emotive phrases such as ‘death sentence’ or sadness. Most felt that diabetes significantly impacted on their daily lives, in terms of dietary restrictions, blood sugar testing, exercising, remembering to take their medications and attending doctor’s appointments. A number of participants share their experiences:

It is sad that I have to follow this routine for the rest of my life.

I feel a little bit sad because disease cannot be cured.

I heard that having diabetes is like under the death sentence.

Test blood sugar many times a day.

Eating and drinking must be limited. Fatty and sweet food must be avoided.

At the same time, participants were proactive at controlling their disease, which included modifying their diet, restricting their consumption of certain foods, exercising and seeking advice on the best way to control blood sugar levels. Many used a trial and error approach to work out the effect of certain foods, and in the future, avoided foods that caused a spike in blood sugar. Other strategies included being prepared for hypoglycaemic episodes and making the most of doctors’ visits to ask questions and to make sense of their medications, referrals and other requirements. Some illustrative quotes follow:

*If I eat delicious food today, tomorrow I will have a strict diet. That is my secret. If I eat delicious food today, next morning, I will test my blood sugar. If its value is 6., that is good. If it is 8., that is not good. I mean the food that night was not good and I won’t try it anymore. I often go dancing on weekends, sweating so much*.

I have no diet, but not eating so much. For che, I only eat half bowl. Previously, I ate so much, 500 g of beefsteak, now only 200 g. I eat red rice and white rice… For exercise, if you are not determined to do your best, you cannot do. Exercise until exhaustion is not good. Swimming is very good and I do 30 – 45 minutes every day.

Participants valued what they considered expert advice, such as advice provided by doctors, diabetes educators and nurses, and relied on this information to make decisions on how to care for themselves. Most were dismissive of other sources of information and had absolute trust in the doctor’s advice, as below:

...if there’s anything that I want to know, I will ask my doctors.

Patients must follow the doctor’s guide carefully, do exercise and go swimming. If not, they will have complications of eyes, heart and kidneys.

We have Vietnamese traditional medicine, but what is the evidence? I never listen to anyone except the doctor.

Although participants were generally satisfied with the doctor’s approach to information sharing, this was not a universal finding. Participant 6, for example, was distressed to realise that a lack of understanding led her to lose out on dental benefits, as below:

The doctor did not inform me clearly, so that the dentist cheated me. We must ask, the doctor will talk… If we do not ask, the doctor won’t explain or guide. Family doctors, nurses and experts only refer. We want to understand, we must ask.

For example, I went to podiatrist. He often said my feet were good. When I asked him how the change of the foot was, at that time he just explained to me. So if we go to see doctor or health workers, we must ask. If we do not have questions, they won’t talk.

### Staying healthy

In theme 3, participants were unanimously motivated to take care of their diabetes, based on their concerns for the future and a desire to stay as healthy as possible. Concerns included avoiding complications such as heart, kidney and eye disease primarily. Some participants were especially motivated by the experiences of acquaintances, who had developed diabetes complications. The following quotes demonstrate these concerns:

I have a friend with diabetes…one leg was amputated then 4 years later the other leg and after 6 months he died. That is my most fear, so I must take care of myself by diet and exercise.

I want to live longer with my offspring.

To protect my health.

Participants were also inspired to take care of their diabetes, when they received support and encouragement from diabetes educators and doctors.

Both my doctor and diabetes educator encourage me to take action in managing my diabetes. The educator teaches me the right way to cook and eat foods, and the information is very detailed. … And because I have paid a lot of attention to these aspects during my years adjusting to diabetes, my conditions seems to not have changed.

Despite their dietary and other life restrictions, many of the participants were philosophical and accepting of their disease. These participants found they were able to adapt without too much difficulty as follows:

I don’t feel there is anything too difficult about managing my diabetes – I tend to do some simple exercises as shown on television (channel 31). After I eat breakfast, I like to go for a walk for an hour. I do not find there are any difficulties, neither in making sure I remember to take my medication, nor in using the information services, nor exercising.

There was also a social element to staying healthy and participants were keen to share their own experiences about what worked for them, so that others could do similarly. The also wished to learn from the diet experiences, of other Vietnamese with diabetes, particularly around experimenting with food. Later they adapted this information their own situation and tastes.

My friend with diabetes bought hai sam (sea cucumber) fried with lean pork, and bitter melon for 2–3 times a week. I mimic her and when I check with my doctor, my blood sugar level also goes down.

Advice for individuals newly diagnosed with diabetes included mainly practical advice such as exercising to one’s capacity and maintaining a healthy diet. Some illustrative quotes follow:

You must listen to your doctor, remember to take your medication, and be conscious of what you eat and drink – that means, less salty, sugary and fatty foods, no smoking or alcohol, perhaps consider changing the things you eat to have a lower GI level, for example, eat brown or red rice instead of white rice, and consume less meat. Exercise is also especially important – it doesn’t have to be vigorous, but can be as simple as walking around the block for half an hour or so.

I would advise them, based on my own experiences, about my way of living – as far as eating, exercising and shopping for the right foods… you should exercise to your own strength and abilities: if you’re fitter, you can exercise more and if you’re feeling not so well, exercise a little less.

### Improving services and information access

In the final theme, issues of services and information access are explored. Participants attended a number of healthcare professionals for their diabetes care, including family doctors, medical specialists, diabetes educators, podiatrists, ophthalmologists and dieticians. For the most part, participants were satisfied with the care and information they received. Some suggestions were nonetheless offered for improvement and are included in the three elements of this theme: (1) a desire to be treated with respect; (2) availability of information in their own language; and (3) a request for reminders, prior to appointments and important tests.

In the first case, participants had high expectations of services, and particularly of doctors, and expected to be guided, advised, informed and treated with respect. This also included an unrushed environment. P6 and P4 explain:

Doctors must listen to the patients and check them. Doctors have no right to say we speak wrongly. If we say something wrong, the doctors have duty to explain to us.

Family doctors should have more time to share information with patients, and have information in Vietnamese and have interpreters. Many doctors only work perfunctorily and do not talk with patients.

I think it is the role of the GP to inform the patients of all the service options.

Secondly, participants unanimously wished to access information about their condition in Vietnamese, and although some information was available in their own language, this was not a universal experience. They additionally wished to receive any new information in this medium.

I would like to receive new information in Vietnamese.

But sometimes, it does become a bit of a barrier when the doctors speak too fast. Translated pamphlets help me to understand better.

Finally, participants wished to receive reminders prior to appointments and important tests. This, they felt was necessary to ensure that they did not forget to attend additional appointments.

I want some authorities, who in charge in diabetes are able to control, inform, guide, remind or warn us to prevent complications. By the way, I say thank you to Australian government about humanity.

Reminding letters are very good.

## Discussion

The principal aims of this project were to explore the health consumer experience of access to diabetes services and diabetes self-management in the Inner North West Melbourne region. The perspective is from a particularly hard to reach population, elderly individuals with diabetes and with low English literacy. The project has been successful in this regard and this paper reports on the findings of three focus groups conducted in Vietnamese. Moreover, this study was a qualitative study and the particular approach used, facilitates an understanding of participants’ perceptions of barriers to health care access. This depth of perception is not achievable through quantitative studies, such as surveys. There were however, some limitations to the study and the number of participants (n = 15) was relatively small, as a result of difficulties accessing participants and also the cost of recruitment, conducting focus groups in Vietnamese, and use of interpreters and translating facilities. The exploratory nature of the focus groups and the pre-determined questions are other limitations, as they prohibited more in depth discussion of the experiences and concerns of participants. For the future, we would recommend that focus groups are dedicated to a single focus, such as experience of diabetes self-management or access to services.

Nonetheless, despite these difficulties, a number of useful insights have emerged from the study. Insights include; (1) the participants’ high levels of motivation to stay healthy; and (2) their expectations of health services, which included a request for information and reminders in Vietnamese. Each of these points is discussed below.

### Motivated to stay healthy

Participants in this study were intensely interested in remaining healthy and in taking care of their diabetes, in order to avoid more serious complications. In this regard, they were proactive in seeking resources and information to assist them in their quest, and this finding is contrary to earlier work in this field [37-39]. Mull et al., [37], for example, who explored the experience of type 2 diabetes among Vietnamese migrants in the US, found that as many as 75% of participants failed to achieve good glycaemic results, related to differing cultural understandings of diabetes, a tendency to reduce medication use when feeling ‘out of balance’, and an aversion to insulin use. This humoral notion of diabetes as the manifestation of a body ‘out of balance’ also appears in the work of Shaw et al., [39], and Helman [40], who additionally found that Vietnamese individuals’ poor health literacy and cultural beliefs about diabetes, hindered their understanding and appreciation of the seriousness of the disease. Moreover, elderly Vietnamese migrants are generally considered to be a marginalised group with limited access to services and facilities [23,27,41] and with limited understanding of their condition [37-39]. Social factors which contribute to this situation, include: low socio economic status [23], poorer general education [24] and low English language skills [25].

Participants in our study were from a disadvantaged area, and similar to other studies of Vietnamese migrants, they presented with low educational backgrounds and with limited English language skills [14,25]. Nonetheless, they appeared to be highly motivated to adhere to dietary and exercise principles, compared to other studies of Vietnamese participants [37-39]. It is not immediately clear why this was the case and we sought answers from the literature. However, a thorough search of the literature located just a single US study that concurred with our findings and which might provide some insight. Orzech et al., [42] found that of four ethnic groups (White, Black, Vietnamese and Latino) with type 2 diabetes, Vietnamese participants reported the highest level of adherence to diet and exercise regimes. These authors postulated two possible explanations for their findings: increased food security among Vietnamese migrants, related to food stamps and social security; and a tendency for Vietnamese participants to be less obese, and thus more receptive to suggested increases in exercise, compared to other groups.

In our study, a further possible explanation presents and participants were very concerned about developing complications, such as heart, eye or kidney disease. This fear of complications was identified, almost unanimously by participants, as influencing their decision to take care of their diabetes. Similar findings are noted in the more general literature around diabetes, where fear of complications has been identified as one of the prime motivating factors in making diabetes related behavioural changes [43,44].

### Expectations of health services

In the second instance, participants in our study had expectations of health services that included timely access to services and referral to specialist services when required, and these expectations are echoed in the literature, where timeliness and access to services are identified as among the most important aspects of care [45]. Participants also desired to be treated with respect, to have their questions answered, and to receive information and guidance about how best to manage their condition. Similar concerns about being treated with respect and dignity [46], and having access to information, are voiced by patients, with chronic illnesses, from all cultural backgrounds [46,47]. Other factors such as the doctor’s communication skills and cultural sensitivity are also identified, in the literature, as important in promoting health service access and adherence to health recommendations [48].

Participants, in this study, placed a high level of trust in the doctor and this feature may have contributed to their adherence to health recommendations, as trust in the physician is generally considered to improve adherence to treatment [49]. Other studies found that higher levels of trust in the doctor was also linked to better glycaemic control [50] and less emotional burden for the patient [51].

Most participants identified a need for information about basic services, and day to day management of their diabetes, and generally identified doctors as the group of health professionals they were most likely to contact for information and services. This finding of considerable reliance on doctors is surprising, as at the same time, participants described receiving information from a number of other sources, such as diabetes educators and community centres.

Study participants also identified a need for information and appointment reminders in Vietnamese and this finding is consistent with the low levels of education and low English language proficiency, found in this group. Similar findings of a need for information in their own language was found in other health related studies among Vietnamese health consumers [24,52] while other authors have concurred with the usefulness of reminders to improve appointment attendance among ethnic minority and underserved populations [53,54].

However, although participants reported a desire for Vietnamese language information, there was no mention of cultural sensitivity as an important aspect of care, which is in contrast to other studies of Vietnamese health consumers [37,40,55]. This finding may mean that this aspect of care was not a concern for participants in our study, or perhaps, similar to other studies, it may mean that professional competence and courtesy were deemed more important than the health professional’s awareness of cultural mores [56,57].

### Implications for care

This study has important implications for care, as rates of type 2 diabetes continue to rise in Australia and globally. There is strong evidence to suggest that elderly Vietnamese migrants are particularly at risk of T2D, and of serious complications of the disease, based on ethnicity and social factors such as low educational status, low socio-economic status and low English literacy. To add to this dilemma, this population is also likely to encounter difficulty with health service access and utilisation. As Vietnamese migrants make us a sizeable portion of the population in Australia generally, and in our local area particularly, it is extremely important that strategies are adopted to encourage this group to effectively self-manage their diabetes. Effective self-management will have the effect of reducing the rate of serious complications such as heart, eye, and kidney disease and will reduce the impact of such complications on the individual, on families and on health services.

There is a need for targeted information about type 2 diabetes, which should be available in the Vietnamese language, and in a simple format, to counter low levels of education generally in this community. This information should be available at the doctor’s waiting room, community centres, pharmacies, and venues frequented by the Vietnamese population. Participants in this study additionally identified Vietnamese radio and newspapers as appropriate media for information dispersal and these media could be used to inform individuals of the risk of diabetes, and the importance of diet and exercise and the need for regular health checks. Frequent broadcasts and notices could potentially act as reminders for appointment attendance, as this is an area of deficit recognised by participants.

## Conclusions

Participants in this study were generally happy with the healthcare they received and were highly motivated to care for their diabetes, based principally on a fear of complications. They additionally highlighted a need for access to interpreters and to diabetes related information in Vietnamese. Although ethnicity and social circumstances together impact on service access, good results can be achieve, for this group, by the provision of sympathetic services and information in Vietnamese.

## Competing interests

The authors declare that they have no competing interests.

## Authors’ contributions

MCO contributed to the design of the project, analysed the data and wrote the manuscript. AC was project manager, contributed to the design of the project, analysed the data and commented on the manuscript. RQ provided advice and assistance on the conduct of the language mediated (Vietnamese) focus groups, contributed to the design of the project and commented on the manuscript. SL contributed to the design of the project and commented on the manuscript. All authors read and approved the final manuscript.

## Pre-publication history

The pre-publication history for this paper can be accessed here:

http://www.biomedcentral.com/1472-6963/13/447/prepub
